# Retrospective analysis of lithium treatment: examination of blood levels

**DOI:** 10.3389/fpsyt.2024.1414424

**Published:** 2024-08-30

**Authors:** Tuğçe Uskur, Oya Güven, Mustafa Tat

**Affiliations:** ^1^ Department of Medical Pharmacology, Faculty of Medicine, Kırklareli University, Kırklareli, Türkiye; ^2^ Department of Emergency Medicine, Faculty of Medicine, Kırklareli University, Kırklareli, Türkiye; ^3^ Medical Biochemistry, Kırklareli Training and Research Hospital, Kirklareli, Türkiye

**Keywords:** lithium treatment, retrospective study, drug levels, neuropsychiatric disorders, treatment efficacy, drug interaction

## Abstract

**Introduction:**

Lithium is a key medication for treating various neuropsychiatric disorders, with a narrow therapeutic index and significant drug interactions. Monitoring lithium blood levels is crucial. This study aims to investigate the relationship between lithium blood levels and demographic characteristics such as age and gender, as well as possible drug interactions, in patients with a history of lithium use who applied to various services and outpatient clinics.

**Materials & methods:**

The files of 438 patients who were admitted to various services and outpatient clinics of Kırklareli Training and Research Hospital between January 1 and December 31, 2023, were retrospectively reviewed. Patients’ blood lithium levels, gender, age, service/outpatient clinic they admitted to, other medications used, urea, creatinine, and eGFR values were recorded.

**Results:**

When the demographic characteristics of 438 patients were examined, 62% were female (270), 38% were male (168), and the average age was 46.3 ± 14.8 years, showing a normal distribution. It was found that 192 patients (71 males, 121 females) had therapeutic lithium blood levels, while 244 patients (97 males, 147 females) had levels below 0.6 mmol/L. Two female patients had blood levels above the therapeutic range (1.23 and 1.43 mmol/L). Among the clinics and services, the four most frequented were the psychiatry clinic (314 patients), internal medicine clinic (36 patients), emergency service (27 patients), and medical oncology clinic (17 patients). Of the 314 patients admitted to the psychiatry clinic, 168 had therapeutic drug levels; only 7 of the 36 admitted to internal medicine had therapeutic levels; 12 of the 27 patients in the emergency service had therapeutic levels; and all 17 patients in medical oncology had levels below therapeutic limits.

**Discussion:**

The data emphasize the importance of regular blood level monitoring to ensure lithium treatment’s efficacy and patient safety. It is noteworthy that most patients in the psychiatry clinic had therapeutic drug levels, while those in other clinics had lower levels.

**Conclusion:**

In conclusion, this study highlights the importance of regular blood level monitoring to ensure the efficacy and safety of lithium treatment.

## Introduction

Lithium, a mood-stabilizing medication, has been a first-line treatment for various neuropsychiatric disorders for over 50 years and has opened many doors in psychiatry (1). The use of lithium salts as a medication in medicine dates back 150 years, with its first discovery in the field of psychiatry occurring in 1949 when Cade identified its specific therapeutic effect on manic episodes. Two years later, Noack and Trautner reported its long-term protective effects and ability to prevent disease recurrence (2). Lithium received FDA approval for the treatment of acute mania in 1970 and maintenance therapy in 1974 and has since been widely used in the treatment of bipolar disorder (3). Generally, lithium is used for the treatment of acute mania, maintenance therapy of bipolar disorder, maintenance therapy in recurrent depression, as an augmenting agent in treatment-resistant depression, and in the treatment of neutropenia (4).

Although lithium has been widely used in the treatment of bipolar disorder for over half a century, we still have limited knowledge about its therapeutic mechanism of action (5). Although its exact mechanism of action is not fully understood, it is thought that lithium regulates G proteins in the phosphatidylinositol system through the second messenger system, inhibits inositol monophosphatase enzyme, and regulates the expression of genes for growth factors and neuronal plasticity by inhibiting protein kinase C and glycogen synthase kinase 3 (6). Additionally, lithium is predominantly excreted by the kidneys, with lithium clearance being approximately 20% of creatinine clearance (7). Therefore, clinicians must monitor renal function tests alongside drug levels to ensure safe and effective treatment.

Due to its narrow therapeutic index, clinical use of lithium requires monitoring of blood levels as toxic effects can occur even when blood levels are within therapeutic limits. Common side effects include tremor, nausea, anorexia, diarrhea, polyuria, polydipsia, nephrogenic diabetes insipidus (which can develop within weeks to months), goiter (which typically develops after months to years), hypothyroidism (which can occur after several months of treatment) and weight gain (which can occur within the first few months of treatment) (8, 9). In addition to its narrow therapeutic index and side effects, lithium-drug interactions are crucial considerations in lithium therapy. According to the Drugs.com database, lithium interacts with 715 drugs at minor, moderate, or major levels (10). The most commonly prescribed medications with the potential to interact with lithium include ACE inhibitors, angiotensin II receptor antagonists (sartans), diuretics, and nonsteroidal anti-inflammatory drugs (NSAIDs). When any of these drugs are initiated, lithium concentrations should be monitored more closely because lithium typically leads to pharmacokinetic interactions, resulting in either decreased or increased elimination, which directly affects lithium blood levels (11).

Lithium blood levels can be measured once every 5-7 days when starting treatment and once every 6 months in patients who have been on long-term treatment and have achieved stability in blood levels (12). Lithium blood levels are measured at these intervals due to the pharmacokinetic properties of the drug, which require time to reach a stable concentration in the body. The half-life of lithium varies depending on the formulation. For instance, lithium carbonate, usually in tablet form, has a half-life of approximately 18-24 hours, which may extend with continuous use. Although not available in our country, liquid lithium citrate is absorbed more rapidly but has a similar half-life range. Monitoring every 5-7 days initially helps ensure the drug stays within the therapeutic range and minimizes toxicity risk (13, 14).

The therapeutic range for lithium is generally accepted to be 0.6-1.2 mmol/L (millimoles per liter). However, there is no consensus on this issue and it should be evaluated according to the patient’s characteristics and response to treatment. Some sources consider the therapeutic range wider, between 0.5 and 1.2 mmol/L (15). The recommended concentration in the US is considerably higher than in Europe, with 1.0–1.5 mmol/l for acute mania and 0.6–1.2 mmol/l for long-term treatment (12). Most patients respond to the drug without signs of toxicity within this range. However, the response and side effects of lithium treatment are individual.

This study aims to investigate the lithium blood levels in patients with a history of lithium use who presented to various services and clinics of Kırklareli Training and Research Hospital between January 1 and December 31, 2023.

## Materials and methods

### Data source

In this study, data were obtained by retrospectively scanning the files of 438 patients who presented to various services and clinics of Kırklareli Training and Research Hospital between January 1 and December 31, 2023. The dataset included patients’ lithium blood levels, gender, age, and the name of the service/clinic they presented to, along with other medications used by the patients, and their creatinine and glomerular filtration rate (GFR) values. The inclusion criteria for the study were: (1) patients with documented use of lithium, (2) patients who had at least one documented lithium blood level measurement from their medical records prior to the study initiation, and (3) patients over the age of 18. Exclusion criteria included: (1) patients with incomplete medical records, (2) patients with inconsistent lithium dosage information, and (3) patients under the age of 18.

The study was conducted with the approval of the Ethics Committee of Kırklareli University (No: P202400018R01-09).

### Data handling and outlier management

Access to data and data analysis were conducted by 3 investigators. Rigorous data handling procedures were implemented to ensure data integrity. Records were screened for completeness and relevance. Missing data points led to exclusion from the analysis and outliers were identified using statistical methods (standard deviation and interquartile range) and reviewed individually for validity.

### Lithium determination

Lithium blood levels were measured using the colorimetric method on the Roche Cobas Pro device. Lithium’s therapeutic blood level was 0.6-1.2 mmol/L.

### Drug interaction analysis

Drug interaction analyses were performed using the drugs.com database. Drugs used by patients were investigated, and a list containing drug interactions was obtained. These interactions were classified based on severity as severe, moderate, mild, or no interaction.

### Statistical analysis

Data were analyzed using IBM Statistical Package for the Social Sciences (SPSS) 28.0 software for the statistical analyses of the study. In the descriptive statistics of the data, the mean, standard deviation, median, lowest, highest, frequency, and ratio values were used. The distribution of variables was measured using the Kolmogorov-Smirnov and Shapiro-Wilk tests. The Mann-Whitney U test was used to analyze quantitative independent data with a non-normal distribution. The Chi-square test was used to analyze qualitative independent data. The effect level and cut-off value were investigated using the ROC curve. The effect level was investigated using univariate and multivariate logistic regression.

## Results

In the study, when the distribution of 438 patients with a history of lithium use was examined according to gender, it was found that 61.6% were female (270) and 38.4% were male (168). The average age of these 438 patients included in the study was found to be 46.4 ± 14.8, with the average age being 46.2 for females and 46.6 for males, and it was observed that the distribution was normal (p value: 0.11). The age range for female patients was 18 to 86 years, while for male patients, it was 21 to 86 years (**Table 1**).

**Table 1:** Demographic profile, lithium levels and renal values of the participants.

Parameter	Psychiatric Clinic	Internal Medicine Clinic	Emergency Service	Oncology Clinic	Other Clinics	Total
Sex						
Male	106	14	8	7	33	168
Female	208	22	19	10	11	270
Age Range						
18-25	25	-	2	-	7	34
26-35	60	9	3	1	6	79
36-45	72	6	3	4	5	90
46-55	85	9	7	-	11	112
56-65	56	7	7	7	3	80
>65	16	5	5	5	12	43
Lithium Levels						
Therapeutic Level	168	7	12	-	5	192
Below Therapeutic Level	145	28	15	17	39	244
Above Therapeutic Level	1	1	-	-	-	2
Renal Values						
Ure	24,74	30,25	22,77	36,31	28,23	26,31
Creatinine	0,87	0,92	0,69	0,84	0,84	0,87
eGFR	94,46	90,30	88,55	88,56	93,88	93,58
Clinic						
Psychiatric Clinic	314					
Internal Medicine Clinic		36				
Emergency Service			27			
Oncology Clinic				17		
Other Clinics					44	
**Total**						438

**Figure 1:**
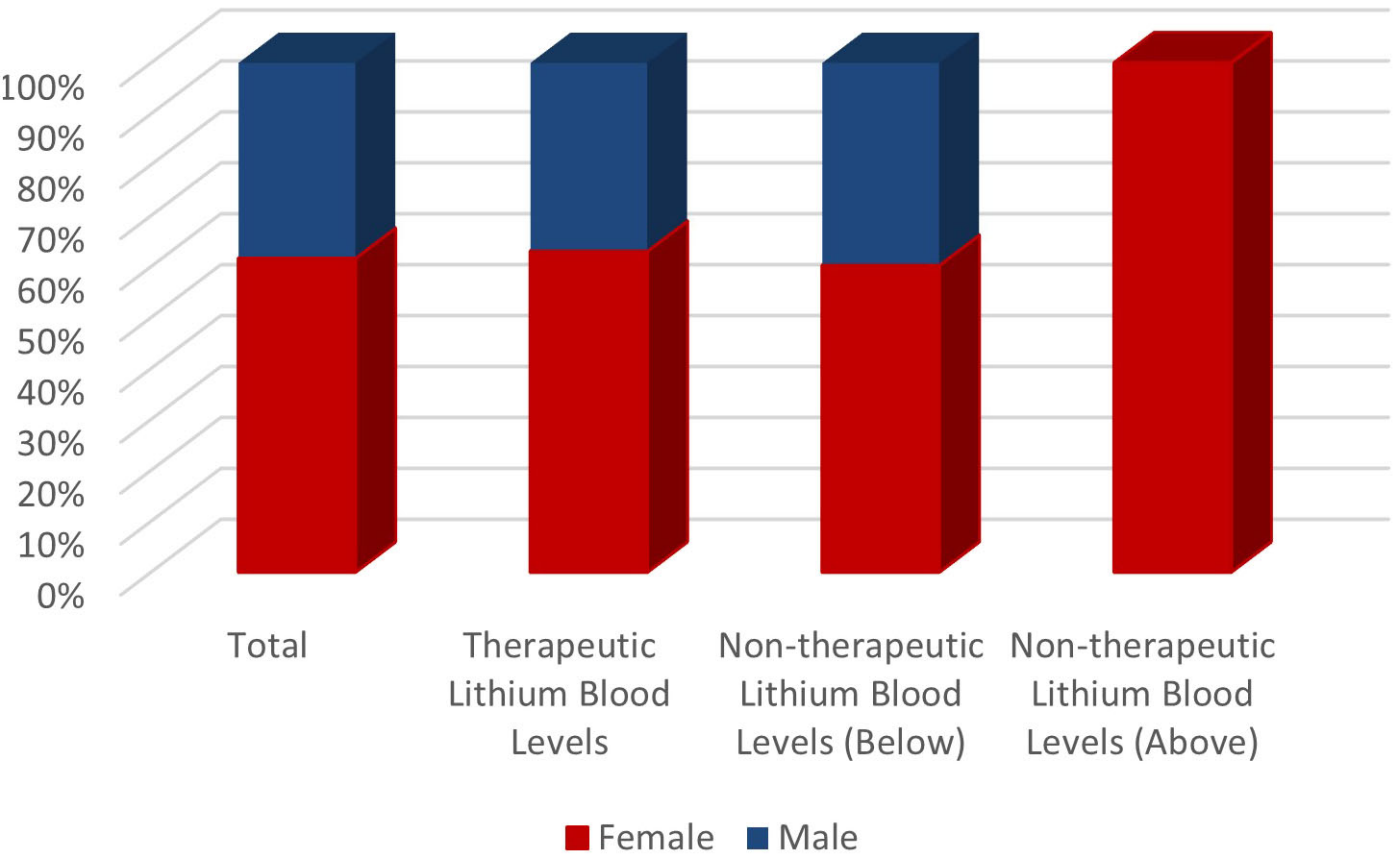
Distribution of lithium levels according to gender.

When the therapeutic blood level of lithium is accepted as 0.6-1.2 mmol/L, it was found that 192 patients had therapeutic lithium blood levels. Of these patients, 71 were male and 121 were female, and the average blood level of the drug was 0.81 (0.5 ± 0.31) mmol/L. While the average drug level of male patients with therapeutic lithium blood levels was 0.46 ± 0.33, it was found to be 0.52 ± 0.34 for female patients (p value: 0.82). It was observed that 244 patients had lithium levels below 0.6 mmol/L, and the average lithium blood levels of these patients were 0.24 mmol/L. Among the patients whose lithium levels were below therapeutic limits, 97 were male, and 147 were female. It was observed that 2 female patients had drug blood levels above the therapeutic range, with values of 1.23 and 1.43 mmol/L, respectively (**Figure 1** and **Table 1**).

**Figure 2:**
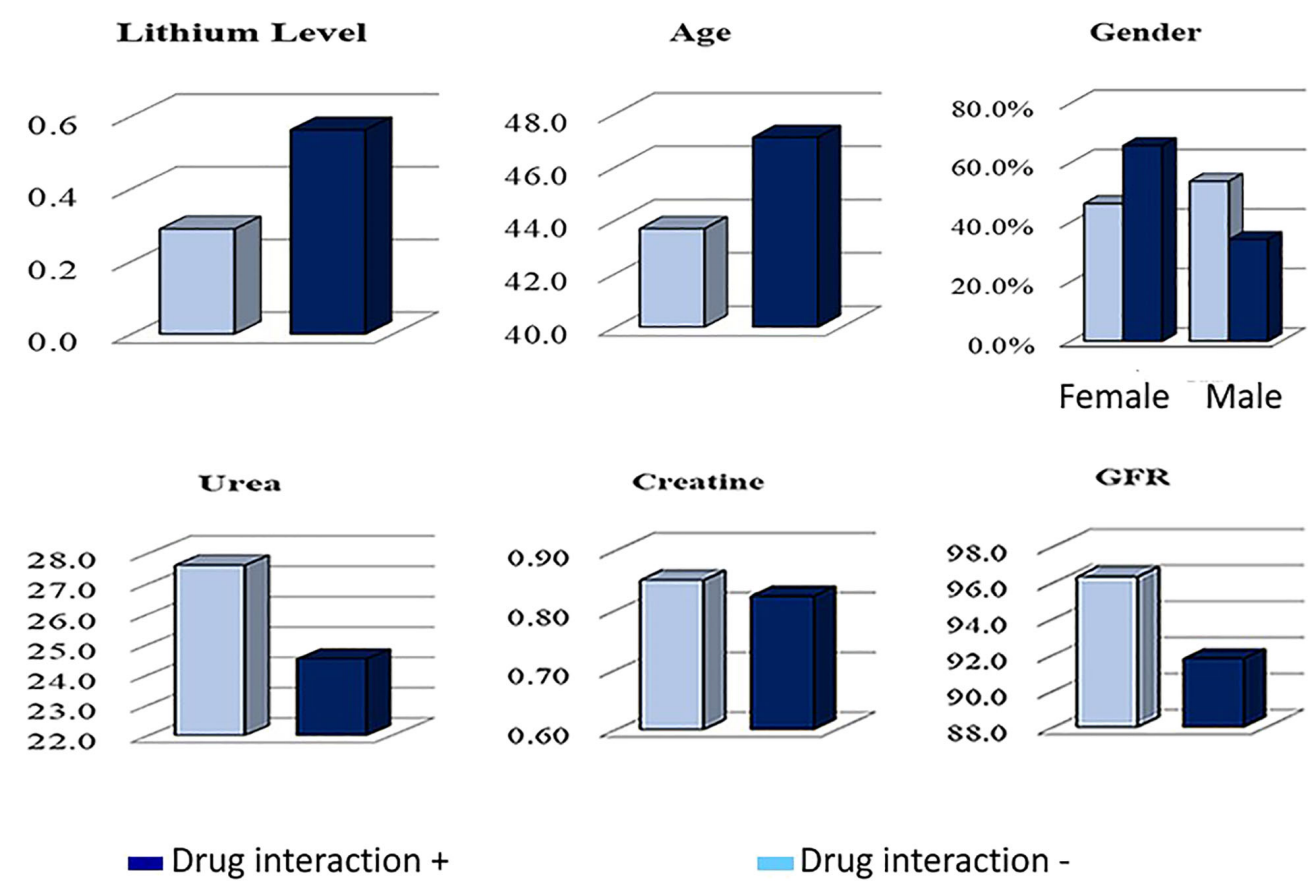
Analysis of lithium-drug interactions: effects of lithium levels, age, gender, and renal parameters.

**Figure 3:**
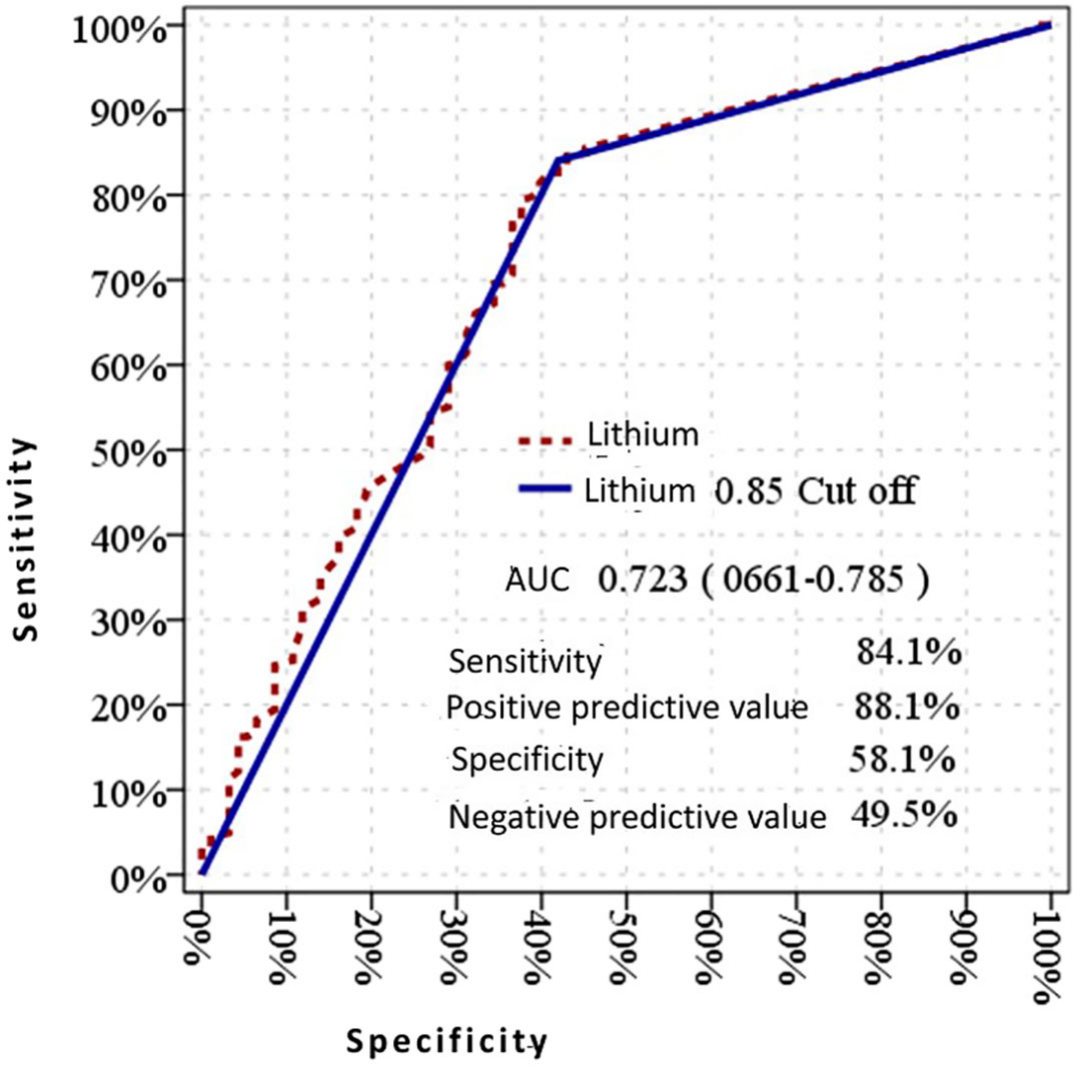
Diagnostic performance of lithium 0.85 mmol/L cutoff for drug interactions: sensitivity, specificity, positive predictive value, and negative predictive value.

Examining lithium-drug interactions among the 244 patients with sub-therapeutic lithium levels revealed interactions with 125 drugs as major, 261 drugs as moderate, and 15 drugs as minor. Gender analysis showed 86 major, 171 moderate, and 12 minor interactions in females, and 39 major, 90 moderate, and 3 minor interactions in males (**Table 2**). It was found that 78.8% of patients using lithium experienced drug interactions, and 71.2% of them had minor to moderate interactions. In the univariate model, significant effects of age, gender, and lithium levels (p<0.05) were observed in distinguishing patients with and without drug interactions, while no significant effect of GFR (p>0.05) was detected. In the multivariate model, age, gender, and lithium levels showed significant independent effects (p<0.05) in distinguishing these patient groups (**Figure 2**). Lithium drug interactions are more prominently influenced by gender and age compared to other findings (**Table 3** and **Figure 3**). The most frequently prescribed drugs interacting with lithium were quetiapine, olanzapine, sertraline, aripiprazole, and venlafaxine. Quetiapine, olanzapine, and aripiprazole exhibited moderate interactions with lithium, whereas sertraline and venlafaxine had major interactions. Notably, drugs like empagliflozin and carbamazepine, known to lower lithium blood levels, were present in the prescriptions. In the two patients with lithium blood levels above therapeutic levels, 4 moderate and 2 major drug interactions were observed, including interactions with zofenopril and hydrochlorothiazide, which increase lithium levels. Among the 192 patients with therapeutic lithium blood levels, there were 105 major, 268 moderate, and 8 minor drug interactions. Gender analysis showed 70 major, 177 moderate, and 7 minor interactions in females, and 35 major, 91 moderate, and 1 minor interaction in males. When patients were divided into two groups based on the presence or absence of drug interactions, it was found that the proportion of women and the average age were higher in the group with drug interactions compared to the other group (**Table 4**). The most frequently prescribed drugs interacting with lithium in these patients were quetiapine, olanzapine, aripiprazole, sertraline, and lamotrigine.

**Table 2:** Lithium - drug interactions.

Lithium Level	Total Patients	Major Interactions	Moderate Interactions	Minor Interactions
**Sub-therapeutic**	244	125	261	15
**- Female**	158	86	171	12
**- Male**	86	39	90	3
**Therapeutic**	192	105	268	8
**- Female**	121	70	177	7
**- Male**	71	35	91	1
**Subra-therapeutic**	2	2	4	-
**- Female**	2	2	4	-
**- Male**	-	-	-	-

**Table 3:** Univariate and multivariate logistic regression analysis results (forward LR).

	Univariate Model					Multivariate Model				
	OR	%95 CI			p	OR	%95 CI			p
Age	1.016	1.000	–	1.032	***0.048***	1.024	1.007	–	1.040	***0.005***
Gender	0.447	0.281	–	0.711	***0.001***	0.444	0.269	–	0.733	***0.001***
Urea	0.979	0.961	–	0.997	***0.023***					
GFR	0.988	0.976	–	1.000	0.051					*** ***

Significant values (p<0.05) are marked in bold.

**Table 4:** Comparison of demographic and clinical parameters between patients with and without drug interactions.

		Drug Interaction (-)				Drug Interaction (+)				p	
		Mean ± SD (n - %)			Median	Mean ± SD (n - %)			Median		
Age		43.7	±	14.7	42.0	47.1	±	14.8	47.0	***0.029***	^m^
Gender	Female	43		46.2%		227		65.8%		***0.001***	^X²^
	Male	50		53.8%		118		34.2%			
Lithium		0.29	±	0.33	0.04	0.56	±	0.32	0.59	***0.000***	^m^
Urea		24.5	±	10.7	22.0	27.6	±	13.1	24.0	***0.040***	^m^
Creatine		0.85	±	0.29	0.79	0.82	±	0.30	0.78	0.385	^m^
GFR		96.3	±	20.1	97.0	91.8	±	19.7	90.0	***0.043***	^m^

^m^Mann-whitney u test/ ^X²^Chi-square test. Significant values (p<0.05) are marked in bold.

In our study, when the clinics and services visited by 438 patients with a history of lithium use were examined, it was found that the top four clinics or services with the highest number of visits were, as expected, the psychiatry clinic with 314 patients (71.6%), followed by the internal medicine clinic with 36 patients (8.2%), 27 patients (6.2%) visited the emergency service and 17 patients (3.8%) visited the medical oncology clinic (**Table 1**). When the lithium blood levels were examined in these three clinics with the highest number of visits, it was found that among the 314 patients who visited the psychiatry clinic, 168 had therapeutic drug levels, 145 had levels below the therapeutic drug level, and 1 patient had a level above the therapeutic drug level with a value of 1.23. Of the 36 patients who visited the internal medicine clinic, only 7 had therapeutic lithium levels, 28 had lithium levels below 0.6 mmol/L, and 1 patient had a lithium level of 1.43 mmol/L. In the emergency service, 15 patients had lithium levels below 0.6 mmol/L, while 12 patients had therapeutic lithium levels. It was found that the lithium levels of all 17 patients who visited the fourth most visited clinic, the medical oncology clinic, were below therapeutic limits.

In the psychiatry clinic, 106 of the 314 patients were male, with an average age of 45.01 ± 13.65 years. Among the male patients, 47 had sub-therapeutic lithium levels (average lithium blood level: 0.33 ± 0.19 mmol/L) with an average age of 43.75 ± 15.50 years, and they had 3 minor, 64 moderate, and 26 major drug interactions. In contrast, 59 male patients had therapeutic lithium levels (average lithium blood level: 0.78 ± 0.14 mmol/L) with an average age of 45.46 ± 12.56 years, experiencing 2 minor, 86 moderate, and 30 major drug interactions. Among the 208 female patients in the psychiatry clinic, the average age was 43.4 ± 14.05 years. Of these, 98 had sub-therapeutic lithium levels (average lithium blood level: 0.33 ± 0.18 mmol/L) with an average age of 41.52 ± 16.65 years, and they had 5 minor, 111 moderate, and 57 major drug interactions. For the 109 female patients with therapeutic lithium levels (average lithium blood level: 0.82 ± 0.15 mmol/L), the average age was 44.86 ± 12.32 years, with 5 minor, 158 moderate, and 59 major drug interactions.

In the internal medicine clinic, a total of 36 lithium-using patients were seen, comprising 14 males and 22 females. The average age of the 14 male patients was 43.7 ± 13.25 years. Among these males, 11 had sub-therapeutic lithium levels (average lithium blood level: 0.12 ± 0.17 mmol/L) with an average age of 44.21 ± 14.24 years, experiencing 4 moderate and 3 major drug interactions. Only 3 male patients had therapeutic lithium levels (average lithium blood level: 0.85 ± 0.20 mmol/L) with an average age of 41.33 ± 8.73 years, experiencing 3 moderate and 2 major drug interactions. The 22 female patients in the internal medicine clinic had an average age of 53.30 ± 14.43 years. Of these, 17 had sub-therapeutic lithium levels (average lithium blood level: 0.17 ± 0.21 mmol/L) with an average age of 50.33 ± 15.9 years, and they experienced 3 minor, 18 moderate, and 5 major drug interactions. Four female patients had therapeutic lithium levels (average lithium blood level: 0.89 ± 0.17 mmol/L) with an average age of 56 ± 9.23 years, experiencing 2 moderate and 11 major drug interactions.

In the emergency service, a total of 27 patients were attended to, with 8 being male and 19 female. The male patients’ average age was 57.87 ± 12.86 years. Among the male patients, 4 presented sub-therapeutic lithium levels (average lithium blood level: 0.07 ± 0.06 mmol/L) with an average age of 63.3 ± 8.58 years and experienced 2 moderate and 4 major drug interactions. Additionally, 4 male patients exhibited therapeutic lithium levels (average lithium blood level: 0.89 ± 0.23 mmol/L) with an average age of 52.25 ± 15.1 years, and they experienced 4 moderate and 2 major drug interactions. Of the 19 female patients, the average age was 49.36 ± 14.82 years. Among these females, 11 showed sub-therapeutic lithium levels (average lithium blood level: 0.26 ± 0.2 mmol/L) with an average age of 52.27 ± 14.36 years and experienced 1 minor, 13 moderate, and 8 major drug interactions. Furthermore, 8 female patients had therapeutic lithium levels (average lithium blood level: 0.81 ± 0.09 mmol/L) with an average age of 45.37 ± 15.36 years and experienced 1 minor, 12 moderate, and 7 major drug interactions major drug interactions. 8 female patients had therapeutic lithium levels (average lithium blood level: 0.81± 0.09 mmol/L) with an average age of 45.37± 15.36 years, experiencing 1 minor, 12 moderate and 7 major drug interactions.

In the oncology clinic, of the 17 patients, 7 were male and 10 were female, and all had sub-therapeutic lithium levels. The average lithium blood level for the 7 male patients was 0.04 mmol/L, with an average age of 59.28 ± 15.62 years, experiencing 6 moderate and 3 major drug interactions. The 10 female patients had an average lithium blood level of 0.074 ± 0.1 mmol/L and an average age of 55.8 ± 12.09 years, experiencing 1 minor, 7 moderate, and 6 major drug interactions.

Examining the renal parameters of the patients, including urea, creatinine, and GFR levels, the average values for the 192 patients with therapeutic blood lithium levels were 24.24 ± 8.07 mg/dL for urea, 0.88 ± 0.26 mg/dL for creatinine, and 91.89 ± 22 mL/min/1.73 m² for GFR. For the 244 patients with sub-therapeutic blood lithium levels, the average values were 27.82 ± 13.22 mg/dL for urea, 0.85 ± 0.29 mg/dL for creatinine, and 95.19 ± 22.64 mL/min/1.73 m² for GFR. Among the two patients with lithium levels above the therapeutic range, one had a lithium level of 1.26 mmol/L, with urea at 17, creatinine at 0.82, and GFR value at 85 mL/min/1.73 m². The other patient, with a lithium level of 1.43 mmol/L, had a urea level of 57, creatinine at 1.47, and GFR at 38 mL/min/1.73 m². When patients were grouped based on the presence or absence of drug interactions, the urea value was higher and the GFR value was lower in the group with drug interactions compared to the other group (p<0.05). There was no significant difference in creatinine values between the groups with and without drug interactions (p>0.05) (**Table 4**). When grouped by the degree of interaction, there were no significant differences in lithium, urea, and GFR values between the moderate and severe interaction groups (p>0.05). However, in the group with severe drug interactions, the creatinine value was significantly higher compared to the other group (p<0.05) (**Table 5**) (**Figure 3**).

**Table 5:** Comparison of demographic and renal parameters based on degree of drug interaction.

		Degree of Interaction								p	
		Moderate				Major					
		Mean ± SD (n - %)			Median	Mean ± SD (n - %)			Median		
Age		46.2	±	15.5	46.0	48.0	±	14.0	49.5	0.171	^m^
Gender	Female	108		64.7%		119		66.9%		0.669	^X²^
	Male	59		35.3%		59		33.1%			
Urea		23.5	±	9.8	22.0	25.4	±	11.5	22.0	0.136	^m^
Creatine		0.78	±	0.21	0.76	0.87	±	0.35	0.80	***0.049***	^m^
GFR		95.1	±	15.9	90.0	88.6	±	22.3	90.0	0.059	^m^

^m^Mann-whitney u test/ ^X²^Chi-square test. Significant values (p<0.05) are marked in bold.

## Discussion

Lithium is a mood-stabilizing medication that has been the first choice for the treatment of various neuropsychiatric disorders for over 50 years, opening many doors in psychiatry and having a well-defined efficacy-side effect profile (16). Although lithium has been used for many years, and its effectiveness in the treatment of various mental disorders is undeniable, it is a drug that has no effect when it falls below a certain level in the blood and has narrow toxic limits (17). Therefore, regular monitoring of blood levels is required to ensure efficacy while avoiding toxic doses during treatment.

Lithium is a mood stabilizer that is commonly used in psychiatric disorders and is well tolerated by many patients, although it has a narrow therapeutic range (18). Due to the close relationship between the side effects that occur during lithium use and the plasma level, careful monitoring of lithium serum concentration is necessary. In this context, the retrospective analysis we conducted on 438 patients with a history of lithium use in this study is important.

According to the demographic data obtained, 168 of the patients in our study are male, and 270 are female. Lithium treatment was more frequently administered to women in our study. Similarly, a comprehensive study published recently in the literature, which included approximately 1,500,000 patients in Australia in 2022, suggested that lithium was prescribed to women more than men (0.33% vs. 0.30%), as found in our study (19).

On the other hand, lithium, one of the most effective drugs for mood disorders, is approved for use in both children and adults and is currently used from the age of 12 through to old age (20). In our study, the age range of the 438 patients included is between 18 and 86 years, with a mean age of 46.32, demonstrating a normal distribution. Specifically, the average age for women is 46.22, while for men it is 46.65. As a result of the analyses, no significant relationship was found between the patients’ lithium blood levels and their ages. Additionally, a study published by Fung et al. in 2023 suggested no correlation between lithium blood levels and age, which is consistent with our data (21). Conversely, another study published in the same year showed a relationship between low serum lithium concentration and increasing age, contrary to our findings (22). The literature on the effects of age on lithium treatment suggests that pharmacokinetics of lithium may change in elderly patients, particularly due to decreased renal function, making them more prone to side effects (23). The discrepancies between other researches in the literature and our findings may be attributed to different reference ranges chosen for therapeutic lithium blood levels, individual patient differences, chronic illnesses, and concomitant medications.

While 121 of the 270 female patients using lithium have therapeutic lithium levels, 147 have lithium levels below therapeutic limits, and 2 female patients have lithium levels above therapeutic limits. Among male patients, 71 have therapeutic lithium levels, while 97 have non-therapeutic lithium levels. The average drug level of male patients with therapeutic lithium blood levels was 0.46 ± 0.33, while for female patients it was 0.52 ± 0.34 (p value: 0.82). The lack of a significant difference in gender distribution between male and female patients is consistent with the findings of a study conducted by Suganya and Ummar in 2017 (24). Additionally, data from another previously published study, which included 1,548 adults treated with lithium for an average of 38.6 months (SD=30.5) across 17 studies, suggested similar weighted response rates to lithium in 1,043 women (65.6% [N=684]) and 505 men (61.0% [N=308]) (25). Due to physiological and genetic differences between genders, pharmacokinetic differences may arise. This is particularly important for drugs related to central nervous system (CNS) activity. A recent integrated analysis and review of pharmacokinetic differences between genders for CNS-related drugs has shown that women often have higher *in vivo* exposure to many CNS drugs (26). In other words, certain drugs tend to be present at higher levels in women’s bodies compared to men’s. Indeed, in our study, the mean therapeutic lithium level in female patients was higher than in males, although not statistically significant. In lithium treatment, statistically significant differences in drug response based on physiological differences between genders can also be expected. Although our study findings are consistent with the findings of Suganya and Ummar and Viguera et al., gender differences in lithium therapeutic levels could be expected due to pharmacokinetic differences arising from physiological differences in men and women. Variations in the methodology, such as differences in the criteria for therapeutic lithium levels, patient adherence to medication, and monitoring practices, could contribute to the observed differences. Furthermore, individual patient factors, such as comorbidities, concurrent medications, and lifestyle factors, may have influenced lithium levels and response in our study population. The relatively smaller sample size and the specific timeframe of our study may have introduced variability that affected our results.

Lithium, with its narrow therapeutic index and many side effects, is also significantly susceptible to numerous drug interactions, requiring careful monitoring of serum concentrations (9, 11). When we examined the results of lithium-drug interactions, we found that a striking number of drugs interacting with lithium were prescribed to patients with therapeutic, sub-therapeutic, or supra-therapeutic lithium blood levels. For instance, in our study, patients with sub-therapeutic lithium levels were often treated with drugs such as empagliflozin and carbamazepine, which may be responsible for the sub-therapeutic lithium concentrations. In contrast, the prescriptions of patients with elevated lithium levels included drugs like zofenopril and hydrochlorothiazide, which can increase lithium concentrations above therapeutic levels. Additionally, when patients were divided into two groups based on the presence or absence of drug interactions, it was found that the group with drug interactions had a higher proportion of women and a higher average age compared to the group without drug interactions, paralleling the increase in polypharmacy conditions as women age (27). These findings underscore the importance of carefully monitoring drug interactions in patients undergoing lithium therapy, especially in older women, and highlight the need to raise awareness among healthcare providers about these interactions.

In our study, which examined the lithium levels of patients visiting psychiatry, internal medicine, and medical oncology clinics, it was observed that most visits were made to the psychiatry clinic. The majority of patients visiting the psychiatry clinic (53.5%) were found to have therapeutic lithium levels, while the majority of patients visiting internal medicine (77.7%), emergency service (55.5) and oncology (100%) clinics had lithium levels below therapeutic levels. The higher prevalence of therapeutic lithium levels among patients in the psychiatry clinic may indicate better adherence to treatment protocols and/or more careful management of lithium therapy by psychiatrists compared to patients in other clinics. These findings suggest that pharmacokinetic and pharmacodynamic factors, such as drug interactions and patient compliance, may be important when evaluating the potential reasons for the lower lithium levels in patients visiting internal medicine and oncology clinics. Considering the pharmacokinetics and interaction potential of lithium, changes in plasma levels of lithium can be observed as a result of possible interactions with other drugs (28). Particularly in the oncology clinic, where all patients had sub-therapeutic levels, it indicates that these patients require special attention in lithium therapy. The lithium used by oncologists to reduce the incidence of infections in neutropenic patients due to chemotherapy, and its potential interaction with chemotherapeutic agents, could explain the insufficient lithium levels. These findings emphasize the importance of carefully monitoring drug interactions in patients undergoing lithium therapy and tailoring treatment plans to individual patient characteristics. Raising awareness among healthcare providers about these interactions is crucial for improving treatment outcomes and reducing the risk of side effects. When examining the relationship between lithium serum levels and renal function parameters, we found that patients with therapeutic lithium levels had normal renal function values, including urea, creatinine, and GFR levels. In patients with sub-therapeutic lithium levels, although urea levels were slightly higher compared to the therapeutic group, creatinine and GFR values generally remained within normal ranges. This indicates that in these patients, medication adherence and treatment efficacy should be evaluated. Among the two patients with lithium blood levels above the therapeutic range, one patient (a 62-year-old female from the internal medicine clinic with a lithium blood level of 1.43) had significantly elevated urea and creatinine levels, and a markedly reduced GFR, indicating that renal function was severely affected. This suggests that lithium at toxic levels can damage renal function. When patients were divided into two groups based on the presence or absence of drug interactions, the group with drug interactions had higher urea levels and lower GFR levels compared to the group without drug interactions (p<0.05). When patients who developed drug interactions with lithium were divided into groups according to the degree of interaction, the group with severe drug interactions had significantly higher creatinine levels than the other group (p<0.05). These data support the view that lithium, which already has harmful effects on the kidneys, can cause more serious damage to the kidneys when involved in drug interactions (29).

Our study presents significant findings regarding the efficacy and safety of lithium therapy. Demographic factors such as age, gender, and renal function can affect the pharmacokinetics and pharmacodynamics of lithium and should be considered when developing treatment plans. The findings indicate better adherence to treatment protocols in psychiatry clinics, resulting in higher therapeutic lithium levels. In contrast lower lithium levels in internal medicine, emergency service and oncology clinics suggest that drug interactions and patient compliance need careful evaluation. By examining the relationship between lithium serum levels and renal function parameters, we observed that toxic levels of lithium could harm renal function. These findings highlight the critical importance of lithium monitoring not only in psychiatry but also in oncology, emergency service and internal medicine clinics. In this context, increasing physician awareness of therapeutic drug monitoring is crucial for improving treatment outcomes and reducing the risk of side effects. Our study has some limitations due to its retrospective nature. Firstly, we could not directly assess patient compliance and relied solely on existing medical records for analysis. Additionally, we could not fully control for the effects of other health conditions and medications used by patients during the data collection process. These limitations may affect the generalizability of the results. Future studies designed prospectively are recommended to address these limitations and obtain more definitive results.

This study contributes valuable insights to the existing psychopharmacology literature by highlighting the necessity of individualized treatment plans and careful monitoring of drug interactions in lithium therapy.

## Conclusion

In conclusion, regular monitoring of blood levels is important for the effectiveness and safety of lithium treatment, and a deeper examination of patient compliance with medication therapy and drug interactions could contribute to the determination of more effective treatment strategies in clinical practice. The data obtained from this study will provide a deeper understanding of patients’ demographic characteristics and blood drug levels in lithium treatment and raise awareness about lithium-drug interactions and patient compliance with lithium therapy.

## Data Availability

The raw data supporting the conclusions of this article will be made available by the authors, without undue reservation.
